# The measurement of lung volumes using body plethysmography and helium dilution methods in COPD patients: a correlation and diagnosis analysis

**DOI:** 10.1038/srep37550

**Published:** 2016-11-23

**Authors:** Yongjiang Tang, Mingke Zhang, Yulin Feng, Binmiao Liang

**Affiliations:** 1 Department of Respiratory and Critical Care Medicine, West China Hospital of Sichuan University, Chengdu, Sichuan 610041, China; 2 Department of Hospital Internal Medicine, Northwest A&F University, Yangling, Shanxi 712100, China

## Abstract

Chronic obstructive pulmonary disease (COPD) is a chronic airway disease characterized by persistent airflow limitation. Moreover, lung hyperinflation evaluated by lung volumes is also the key pathophysiologic process during COPD progression. Nevertheless, there is still no preferred method to evaluate lung volumes. For this study, we recruited 170 patients with stable COPD to assess lung volumes stratified by airflow limitation severity. Lung volumes including residual volume (RV) and total lung capacity (TLC) were determined by both body plethysmography and helium dilution methods. The discrepancies between these two methods were recorded as ΔRV%pred, ΔTLC%pred, and ΔRV/TLC. We found that ΔRV%pred, ΔTLC%pred, and ΔRV/TLC increased significantly with the severity of COPD. The differences of lung capacity between these two methods were negatively correlated with FEV_1_%pred, and diffusing capacity for carbon monoxide (D_L_CO%pred). Moreover, the receiver operating characteristic (ROC) for ΔTLC%pred to distinguish severe COPD from non-severe COPD had an area under curve (AUC) of 0.886. The differences of lung volume parameters measured by body plethysmography and helium dilution methods were associated with airflow limitation and can effectively differentiate COPD severity, which may be a supportive method to assess the lung function of stable COPD patients.

Chronic obstructive pulmonary disease (COPD) is a disease characterized by persistent and progressive airflow limitation. Spirometric measurements of decreased forced expiratory airflow are the prerequisite in establishing a diagnosis of COPD and classification of airflow limitation^1^,^2^,^3^. However, studies have revealed that simple spirometry may not be sufficiently sensitive to evaluate the diagnosis and severity of COPD, leading to the consideration of other lung physiologic parameters in assessing COPD severities^4^,^5^. Besides airway obstruction, the changes of lung parenchymal structure, resulting in lung hyperinflation (air trapping) and gas exchange abnormalities, are also the key pathophysiologic processes during COPD progression. The corresponding functional studies suggest that lung volumetric parameters such as residual volume (RV) and total lung capacity (TLC) are important measurements in evaluating COPD severities and treatment responses^6^,^7^. However, a gold standard method to evaluate lung volumes has not yet been confirmed.

Both gas (helium) dilution and whole-body plethysmography (WBP) are common methods to measure lung volume. When lung function is normal, there is no difference of lung volume values measured by these two methods. However, in the setting of airflow obstruction, the values measured by these two methods are heterogeneous^8^,^9^,^10^. The guideline of American Thoracic Society/European Respiratory Society (ATS/ERS) on lung volume measurements has not provided a clear statement on recommending one specific technique over the other^6^. However, WBP is commonly used to measure lung volumes especially RV, but may overestimate RV due to the gas within all regions of the lung and airways undergoing unequal and asynchronous compression or decompression during panting maneuvers and excessive compliance of the extrathoracic airway^11^,^12^. Multi-breath Helium dilution method (MBHD) is an alternative method for measuring alveolar volume, but may cause underestimation for the uneven distribution of ventilation and the gas contained within the poorly ventilated regions not incorporating in the helium estimate of lung volume^8^,^9^. As the biases of these two methods are both associated with a degree of airflow limitation, the differences between these two methods may provide an alternative marker to reflect the degree of airway obstruction and be an optimal substitute to evaluate the severity of COPD.

Based on current evidence of lung volumetric parameters in COPD and potential estimation biases in WBP and MBHD methods, we conducted a prospective correlation and diagnosis analysis to further assess the value of lung volume as well as the differences between these two methods in distinguishing COPD severities, to clarify the influences of airflow limitation on lung volume measurement, and to evaluate the correlation with diffusing capacity.

## Results

**Figure 1:**
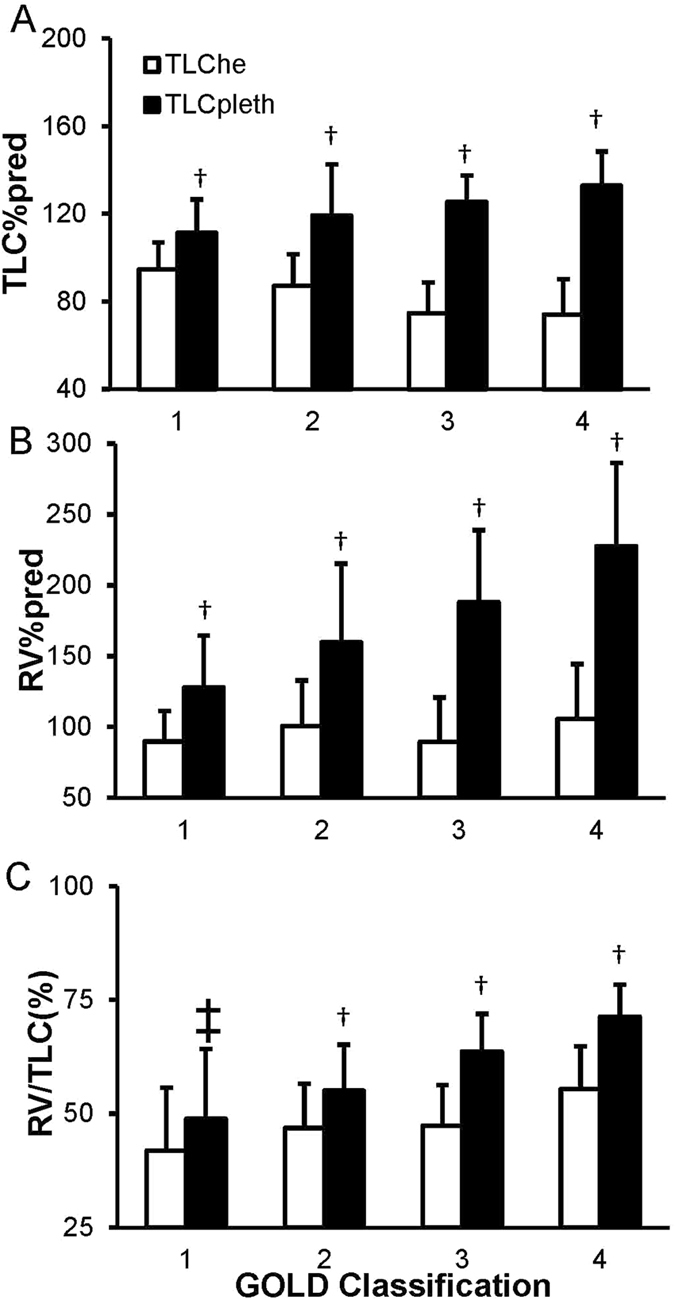
Average TLC%pred (**A**), RV%pred (**B**), and RV/TLC (**C**) as measured by whole-body plethysmography and multi-breath helium dilution, varying by severity. The results are plotted as means ± SD; Differences between body plethysmography and helium dilution methods were analyzed with paired T-test. ^†^*p* < 0.001, ^‡^*p* < 0.05. he, helium dilution; pleth, plethysmography; TLC, total lung capacity; RV, residue volume.

A total of 170 patients were included into this prospective study. All patients were confirmed with stable COPD according to GOLD standards^1^. None of the patients had an acute exacerbation during previous 4 weeks before pulmonary function tests, and all medications which may alter lung function were stopped for at least 72 hours. Pulmonary function variables are performed in Table 1 and Fig. 1. The subject population included 44 patients (25.9%) with GOLD Classification 1, 54 patients (31.8%) with GOLD Classification 2, 34 patients (20.0%) with GOLD Classification 3, and 38 patients (22.3%) with GOLD Classification 4.

**Figure 2:**
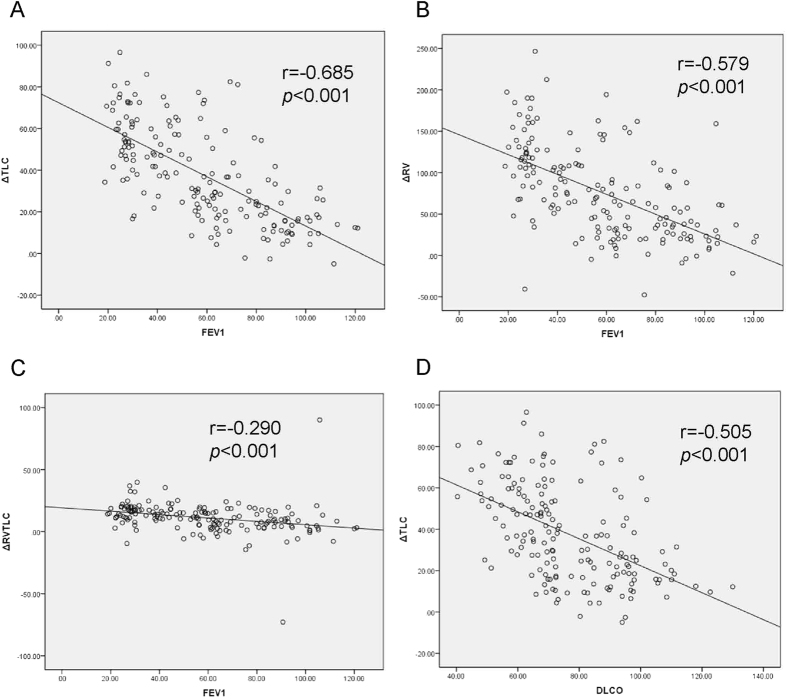
Correlations of the differences of TLC%pred (**A**), RV%pred (**B**), and RV/TLC (**C**) between whole-body plethysmography and multi-breath helium dilution (MBHD) methods, with FEV_1_%pred (FEV_1_). Correlations of the difference of TLC%pred with single breath diffusing capacity for carbon monoxide (D_L_CO) (**D**).

As Fig. 1 and Table 1 shown, TLC%pred, RV%pred and RV/TLC measured by WBP were significantly increased in GOLD Classification 2, 3, and 4, compared with Classification 1. In contrast, these parameters measured by MBHD were no significant difference between different GOLD classifications (Fig. 1). TLC%pred, RV%pred and RV/TLCmeasured by WBP were significant larger than that determined by MBHD in all COPD stages (37.8 ± 22.9%, 75.7 ± 55.0%, and 11.2 ± 12.5%, all *p* < 0.0001, Table 1). The differences of TLC%pred, RV%pred and RV/TLC were significant greater from the patients with GOLD 3 and 4 diseases, compared to the differences from the patients with GOLD 1 and 2 diseases (Table 1). To further confirm the influences of airflow obstruction on lung volume measurement, we studied the correlation of ΔRV%pred, ΔTLC%pred, and ΔRV/TLC with FEV_1_%pred. We found that the difference of TLC%pred measured by these two methods was strong correlated with FEV_1_%pred (r = −0.685, *p* < 0.001, Fig. 2A). There was also moderate but significant correlation between differences of RV%pred and FEV_1_%pred (r = −0.579, *p* < 0.001, Fig. 2B). However, the correlation between differences of RV/TLC and FEV_1_%pred was weak (r = −0.290, *p* < 0.001, Fig. 2C).

**Figure 3:**
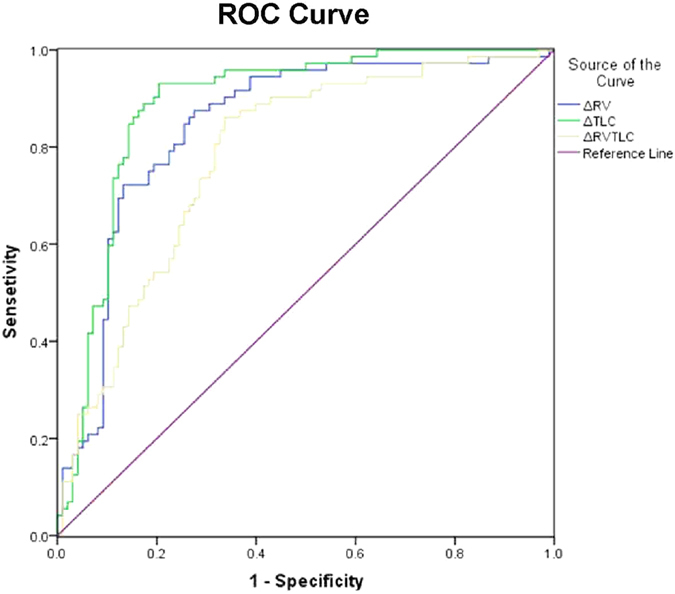
ROC curve for ΔRV%pred, ΔTLC%pred, and ΔRV/TLC in discriminating between mild/moderate and severe/very severe COPD patients, defined by GOLD classification of airflow limitation.

As ΔRV%pred, ΔTLC%pred, and ΔRV/TLC were correlated with FEV_1_%pred, we depicted ROC curves and calculated the area under curve (AUC) to evaluate the accuracy of ΔRV%pred, ΔTLC%pred, and ΔRV/TLC in discriminating different COPD severities. Because of limited patient samples, we combined mild and moderate COPD patients, defined by GOLD classification of airflow limitation, as ‘non-severe’ group; while the severe and very severe COPD patients were combined as ‘severe’ group. We found that ΔRV%pred, ΔTLC%pred, and ΔRV/TLC could efficiently distinguish severe COPD from non-severe COPD with a high AUC (Fig. 3). The most effective variable was ΔTLC%pred with the AUC up to 0.886 (95% CI 0.834~0.939). The ΔTLC%pred value of 34.2 would have a sensitivity of 93.1% and specificity of 79.6%, with the positive and negative likelihood ratio of 4.56 and 0.09, respectively (Table 2). Similarly, as shown in Table 2, ΔRV%pred, and ΔRV/TLC also had a relatively high sensitivity and specificity to differentiate COPD severity.

In our study, we also found that the diffusing capacity measured with diffusing capacity for carbon monoxide (D_L_CO%pred) significantly decreased with increased severity of COPD (Table 1). D_L_CO%pred was significant lower from the patients with GOLD 2, 3, and 4 disease, compared to the patients with GOLD 1 disease (all *p* < 0.05). Interestingly, the difference of TLC%pred as measured by WBP and MBHD was negatively correlated with DLCO%pred (r = −0.505, *p* < 0.05, Fig. 2D), therefore suggesting that this discrepancy may also be associated with the degree of lung parenchymal destruction.

## Discussion

In our study, we found that lung volume variables including TLC%pred, RV%pred and RV/TLC as measured by WBP were significantly increased as COPD exacerbated. These variables measured by WBP were significantly higher than those measured by MBHD method. In addition, differences measured by these two methods were negatively correlated with FEV_1_%pred and effectively differentiated COPD severity. Moreover, we also identified that the discrepancy of TLC measured by these different methods was negatively correlated with diffusing capacity. Thus, lung volume measurement of the differences between these two methods may be an alternative marker to reflect the degree of airflow obstruction and gas trapping.

It has been widely acknowledged that spirometric measurements of FEV_1_ and FEV_1_/FVC are key parameters in diagnosing and grading severity of COPD. However, COPD is a complex disease that is characterized by the presence of airflow limitation, air trapping and emphysema^1^. Decreased FEV_1_ primarily results from small airway obstruction and emphysema^13^. In combination, both airway obstruction and parenchymal destruction lead to expiratory gas trapping, resulting in hyperinflation^1^. Our study found that TLC%pred, RV%pred and RV/TLC measured by WBP significantly increased as airflow limitation worsened, indicating that airflow limitation and gas trapping share the common pathophysiologic change during COPD progression. This suggests that testing of lung volumes could be an effective addition to spirometry in comprehensive assessment of COPD. These additional parameters may help to exclude potential restrictive diseases from obstructive lung disease in patients with dyspnea. Secondly, lung volume is a useful tool to evaluate some COPD properties that is advantageous to FEV_1_. For example, some studies found that an increase of RV/TLC was an independent risk factor of all-cause mortality and frequent exacerbations in COPD population^14^,^15^. Lung volume is also very sensitive to bronchodilators and lung-volume reduction surgery, and better associated with patient-centered outcomes such as dyspnea and exercise tolerance^16^,^17^,^18^. Washko *et al.* found that preoperative RV/TLC ratio but not FEV_1_ is predictive of postoperative outcomes after lung-volume reduction surgery^16^. Thus, integrative analysis of lung functions including both spirometry and lung volume in COPD patients is warranted.

Currently, measurement of lung volumes lacks a gold standard. The guideline of ATS/ERS on lung volume measurements lists out several methods including WBP, MBHD, and imaging techniques^6^. However, the guideline does not make a clear recommendation on which specific technique is the best to use, especially in COPD patients, that lung volumes measured by WBP and MBHD are always not consistent. Consistent with other studies, our study found that TLC%pred, RV%pred and RV/TLC measured in COPD patients by WBP were significantly higher than those measured by MBHD method^8^,^9^,^11^,^19^. Coertjens *et al.* found that in 93 COPD patients (29 mild/moderate, 29 severe, 35 very severe), the differences of TLC between the WBP and MBHD method ranged from 30.5% of the predicted value to 38.2% of the predicted value, which is similar to our finding^8^. O’Donnell *et al.* showed that TLC measured by WBP may be overestimated in COPD patients, as WBP-derived TLC was significant greater than the values measured with MBHD method and CT (0.63 L and 0.87 L, respectively), especially among the patients with FEV_1_ < 30% of predicted^9^. The variations of different methods may be due to the physical principles. Lung volume measured by WBP is based on the Boyle’s law that the product of gas volume and pressure is constant under isothermal conditions. Thus, at any given moment, when a constant amount of gas is compressed or decompressed, the gas volume decreases or increases and gas pressure changes such that the product of volume and pressure^6^. In COPD patients, WBP may overestimate RV with inadequate equilibration of mouth and alveolar pressure. On the other hand, the method of MBHD is based on the equilibration of gas in the lung with a gas containing helium with known volume. As the proportion of poorly ventilated lung units increases, the smaller the values are measured by MBHD. Herein, based on different physical principles between WBP and MBHD, it is reasonable to test the differences of lung volumes by WBP and MBHD in COPD patients.

We further investigated the differences between these two different methods to evaluate the relation between the differences and COPD severity. We found that the differences measured by these two methods were negatively correlated with FEV_1_%pred, and can effectively differentiate severe COPD from non-severe patients. As previous mentioned, underestimation of lung volume by MBHD is associated with the degree of gas trapping. Our findings align with Jarenback *et al.*^19^ in that with single breath or multiple breath helium dilution methods, the helium dilution-derived TLC did not increase based on GOLD classifications. Moreover, O’Donnell *et al.* found FEV_1_%pred <30% is an independent factor for ΔTLC >1L, which also indicates that the difference of lung volumes measured by different methods maybe associated with airflow limitation^9^. Thus, it is reasonable that high sensitivity and specificity of ΔTLC%pred for differentiating severe and very severe COPD from non-severe COPD patients were found in our study. However, the exact diagnostic efficacy of these variables needs further validation with a larger sample population.

D_L_CO is a traditional physiologic marker to assess the potential of the lung for gas exchange. Destruction of alveolar walls in emphysema patients directly disrupts the integrity of alveolar capillary bed, reducing D_L_CO. A pathologic and radiographic correlate of decreased D_L_CO with emphysema was reported that a good correlation between low D_L_CO and decreased total lung tissue volume on chest computed tomography^20^,^21^. In this study we found that diffusing capacity measured with D_L_CO%pred significantly decreased with increased severity of COPD. Moreover, ΔTLC%pred was negatively correlated with D_L_CO, which indicated the potential relationship between airway obstruction and decreased D_L_CO. One study showed that the severity of diffusing capacity impairment is correlated with airway wall thickness, which may induce air trapping^22^. This is further confirmed that increased air trapping induced by metronome-paced tachypnea correlated well with D_L_CO^23^. These findings may raise additional interest in further evaluation of differences in lung capacity between different methods as an index for understanding the structure and function change of COPD.

In addition, our study had several limitations, which may lead to cautious interpretation of the results. First, the sample is small in our study, which may result in bias of our findings. Second, assessment of COPD severity was based purely on spirometry, lacking information regarding exacerbation history and comorbiditiest. Further prospective studies are needed to evaluate the relationship of lung volume with clinical endpoints. In summary, our study indicates that the differences of lung volumes measured by WBD and MBHD in COPD patients are associated with the level of airflow limitation and impaired diffusing capacity, which may be optimal substitute to evaluate the severity of COPD.

## Methods and Materials

### Participants

Outpatients who diagnosed with COPD in West China Hospital of Sichuan University from January 2014 to March 2015 were consecutively enrolled in this study. All included participants met the diagnostic criteria as following: (a) FEV_1_/FVC ratio <0.7 after bronchodilation; (b) no acute exacerbation during previous 4 weeks; (c) stop the medications, which may influence pulmonary function testing for at least 3 days. Participants coexisting medical conditions that would interfere with pulmonary function testing were excluded. This study has been approved by the Institutional Review Board of West China Hospital of Sichuan University, and written informed consents were obtained from all subjects. The methods in this study were carried out in accordance with the approved guidelines.

### Pulmonary function testing

WBP and MBHD method were performed in all enrolled patients by a full MasterScreen PFT System (Jaeger Corp, Germany), which was equipped with a mixing fan, carbon dioxide (CO_2_) absorber, oxygen (O_2_) and helium supply, a gas inlet and outlet, and a water vapor absorber.

WBP measured both lung airflow (FEV_1_, peak expiratory flow (PEF)) and volumes (RV and TLC). All test procedures complied with the standardizations recommended by ATS/ERS guideline^2^,^6^, which contained a series of gentle pants at a frequency between 0.5 and 1.0 Hz to calculate lung volumes, and three distinct phases to depict the flow-volume curves including: (1) maximal inspiration; (2) a “blast” of exhalation; and (3) continued complete exhalation until the volume-time curve showed no change in volume (<0.025L) for ≥1s and the subject had tried to exhale for ≥6s.

Lung volumes (RV and TLC) were also measured by MBHD method according to the following steps: patients were instructed to breathe for 30–60 seconds to achieve a stable end-tidal expiratory level, then switched them to the helium gas (turn in) and noted the helium concentration every 15 seconds until the helium equilibration is complete (i.e. change of helium concentration is <0.02% for 30 seconds), and finally disconnected them from the helium gas (turn out).

Airflow parameters were detected as predicted percentage of FEV_1_ (FEV_1_%pred), PEF (PEF%pred), and maximal mid-expiratory flow (MMEF%pred); while lung volumes were displayed as predicted percentage of RV (RV%pred) and TLC (TLC%pred), and RV/TLC. The predictive equations were adjusted for Chinese subjects^24^. Differences in lung volumes between WBP and MBHD method were calculated as predicted percentage of RV (ΔRV%pred), TLC (ΔTLC%pred) and RV/TLC (ΔRV/TLC).

D_L_CO was measured by Single-breath testing^25^. The subject unforced exhaled to RV, and then inhaled testing gas rapidly to TLC, keeping breath-hold for 10 seconds, and the expiratory gas was collecting for analysis.

### Statistics Analysis

The statistical analysis was performed with SPSS19.0 (SPSS, Inc., Chicago, USA). Normally distributed data were described as mean ± standard deviation (SD). Groups were defined by GOLD classification severity, and differences of the lung volume indexes between WBP and MBHD methods were analyzed with paired T-test. Differences between groups were tested by analysis of variance (ANOVA) and Student’s-Newman-Keuls tests were further used for multiple comparison tests when significant differences among all groups were found. The correlations among these indexes were performed by Pearson correlation analysis. We depicted receiver operating characteristic (ROC) curves and calculated area under the curve (AUC) to evaluate the accuracy of ΔRV%pred, ΔTLC%pred, and ΔRV/TLC in discriminating different COPD severities. Cutoff points were defined as the point when Youden’s index (=sensitivity + specificity − 1) reached the maximum, and the sensitivity, specificity, as well as likelihood ratios (LRs) were also calculated in different severities. A p value < 0.05 was considered statistically significant.

## Additional Information

**How to cite this article**: Tang, Y. *et al.* The measurement of lung volumes using body plethysmography and helium dilution methods in COPD patients: a correlation and diagnosis analysis. *Sci. Rep.* **6**, 37550; doi: 10.1038/srep37550 (2016).

**Publisher's note:** Springer Nature remains neutral with regard to jurisdictional claims in published maps and institutional affiliations.

## Figures and Tables

**Table 1:** Spirometric and lung volume variables in 170 subjects stratified by airflow limitation severity.

Variable	COPD Classification				
	GOLD 1 (*n* = 44)	GOLD 2 (*n* = 54)	GOLD 3 (*n* = 34)	GOLD 4 (*n* = 38)	All Patients (n = 170)
FEV_1_%pred	94.4 ± 10.5	63.4 ± 7.4*	39.8 ± 5.9*^,#^	26.0 ± 3.1*^,#,$^	58.4 ± 26.6
FEV_1_/FVC (%)	63.5 ± 5.6	54.8 ± 9.3*	42.9 ± 7.6*^,#^	34.5 ± 6.1*^,#,$^	50.1 ± 13.1
PEF%pred	80.7 ± 21.4	52.4 ± 14.6*	37.1 ± 11.0*^,#^	24.3 ± 5.6*^,#,$^	50.4 ± 25.4
MMEF%pred	38.0 ± 9.9	22.4 ± 6.8*	14.4 ± 14.0*^,#^	7.3 ± 1.5*^,#,$^	21.4 ± 14.3
TLCpleth%pred	111.4 ± 15.2	119.3 ± 23.4*	125.5 ± 12.0*	132.9 ± 15.5*^,#^	121.5 ± 19.3
RVpleth%	127.8 ± 36.8	159.8 ± 55.4*	188.3 ± 50.5*^,#^	227.6 ± 58.6*^,#,$^	172.4 ± 62.2
RV/TLCpleth (%)	48.8 ± 15.3	55.1 ± 10.1*	63.7 ± 8.2*^,#^	71.3 ± 7.0*^,#,$^	58.8 ± 13.6
ΔTLC%pred	16.7 ± 11.2	32.1 ± 19.9*	50.8 ± 16.0*^,#^	58.8 ± 16.5*^,#,$^	37.8 ± 22.9
ΔRV%pred	38.1 ± 33.2	59.1 ± 50.5*	98.9 ± 45.7*^,#^	122.0 ± 47.0*^,#,$^	75.7 ± 55.0
ΔRV/TLC (%)	7.0 ± 18.7	8.2 ± 8.6	16.3 ± 7.5*^,#^	15.9 ± 8.3*^,#^	11.2 ± 12.5
D_L_CO%pred	90.4 ± 15.8	82.8 ± 16.0*	66.6 ± 4.5*^,#^	59.2 ± 9.5*^,#,$^	76.2 ± 17.8

Data were described as mean ± standard deviation (SD). *p < 0.05 Compared with GOLD classification stage 1; ^#^*p* < 0.05 Compared with GOLD classification stage 2; ^$^*p* < 0.05 Compared with GOLD classification stage 3. FEV_1_, the forced expiratory volume in the first second; FVC, the forced vital capacity; PEF, peak expiratory flow; MMEF, maximal mid-expiratory flow; D_L_CO, single breath diffusing capacity for carbon monoxide; See Fig. 1 for expansion of other abbreviations.

**Table 2:** The value of ΔRV%pred, ΔTLC%pred, and ΔRV/TLC in discriminating between mild/moderate and severe/very severe COPD patients.

Variable	Cutoff Points	Sensitivity	Specificity	LR+	LR−
ΔRV%pred	63.4	0.875	0.724	3.17	0.17
ΔTLC%pred	34.2	0.931	0.796	4.56	0.09
ΔRV/TLC	10.3	0.861	0.663	2.55	0.21

LR, Likelihood Ratio; the severity of COPD was defined by GOLD classification of airflow limitation.
